# Editorial: Biosynthetic pathways and metabolic engineering of bioactive natural products from medicinal plants

**DOI:** 10.3389/fpls.2026.2003419

**Published:** 2026-10-08

**Authors:** Lihang Xie

**Affiliations:** State Key Laboratory of Metabolic Dysregulation & Prevention and Treatment of Esophageal Cancer, Tianjian Laboratory of Advanced Biomedical Sciences, Academy of Medical Sciences, Zhengzhou University, Zhengzhou, China

**Keywords:** biosynthetic pathways, medicinal plants, metabolic engineering, multi-omics integration, secondary metabolism

Medicinal plants are extraordinary biological systems, producing a vast array of structurally diverse natural products with remarkable pharmacological activities. While these bioactive compounds remain indispensable sources for modern drug discovery and therapeutic development, their clinical and industrial applications are frequently bottlenecked by low native accumulation, seasonal variability, and incompletely elucidated biosynthetic mechanisms. To address these limitations, recent advances in multi-omics integration, functional genomics, transcriptional regulation, and synthetic biology have opened unprecedented avenues for high-resolution pathway discovery and rational metabolic engineering. This Research Topic brings together five innovative studies that reflect these cutting-edge strategies, spanning from genome-wide gene family identification and microRNA-mediated flux control to ecological synthesis drivers and artificial intelligence (AI)-driven metabolic modeling.

Glycosylation plays a critical role in altering the solubility, stability, and bioactivity of plant specialized metabolites. Focusing on *Dendrobium huoshanense*, Liu et al. conducted a comprehensive genome-wide identification of the UDP-glycosyltransferase (UGT) gene family. They functionally characterized DhUGT44, demonstrating its pivotal role in mediating flavonoid 7-O-glycosylation and conferring enhanced cold tolerance, directly linking secondary metabolite modification with plant environmental adaptation.

Terpenoids represent one of the largest and most therapeutically valuable classes of secondary metabolites. Studying *Rehmannia glutinosa*, Xiao et al. integrated transcriptomic profiling with Weighted Gene Co-expression Network Analysis (WGCNA). Their work successfully uncovered key candidate hub gene networks governing terpenoid biosynthesis, offering promising genetic targets for future yield optimization and pathway manipulation.

Fine-tuning competitive pathway branches is essential for targeted metabolite production. In Salvia miltiorrhiza, You et al. elucidated the regulatory mechanism of Smi-miR164a. Their findings revealed that Smi-miR164a functions as a dual-switch controller—positively regulating phenolic acid accumulation while downregulating tanshinone production—providing novel insights into microRNA-driven post-transcriptional control of metabolic flux.

Environmental drivers strongly dictate the natural synthesis and metabolic plasticity of phytochemicals. Samarina et al. presented a comprehensive review summarizing the environmental controls governing anthraquinone and flavonoid accumulation in wild plant species. They highlighted how ecological stressors shape biosynthetic pathway evolution and adaptive significance, offering strategic entry points for sustainable wild resource conservation and field-based quality management.

As multi-omics datasets scale exponentially, system-level data interpretation demands advanced computational frameworks. Chen et al. explored the AI-driven integration and optimization of medicinal plant multi-omics metabolic networks. Their study illustrates how machine learning algorithms can accelerate key enzyme discovery, predict metabolic flux, and streamline synthetic biology pipelines for green factory production.

Collectively, the five contributions in this collection highlight the power of combining molecular genomics, ecological insights, and computational tools to decode plant secondary metabolism. By systematically resolving enzymatic steps, regulatory networks, and system-level dynamics, this Research Topic provides a solid foundation for harnessing medicinal plants as sustainable production platforms for high-value bioactive compounds.

